# Miscellaneous Notices

**Published:** 1850-07

**Authors:** 


### Miscellaneous Notices.

### *Ninth Annual Meeting of the American Society of Dental Surgeons, held at Saratoga, August,* 1848.

Reported by William Henry Burr, Phonographer.

First Day, 3½ P. M.

The Society assembled to discuss the subject for the present year, which was "Filling Teeth."

The conversation was opened by Dr. Amos Westcott, who suggested that first in order of the discussion they should take up that part relating to *filing and otherwise separating* the teeth for filling. The suggestion, meeting the approbation of the meeting, he proceeded to state his method of practice in this particular. He said (speaking particularly of front teeth) that he used the file pretty freely, and did not now think of but one exception to the use of it, and that was where the teeth approximate so as to come quite together at their points, and not at the gum. In such cases it was his practice to use wedges.

Dr. C. A. Harris said that he had taken some pains to inform himself with regard to a practice which had obtained among many eminent practitioners, that of separating the teeth by means of wedges of wood, or the interposition of some other substance. Previously to the completion of the growth of the body it may be done in most cases with impunity. The operation may often be performed with safety after the age of fifteen or twenty, or even twenty-five or thirty, but as a general rule, it should not be attempted earlier than the fifteenth or later than the twenty-fifth or thirtieth year. When performed earlier or later, it is apt to produce inflammation in the alveolo dental periostal membrane and gums, which, ever after, renders them more liable to disease. A gentleman of the profession some six years ago, called on me to obtain my opinion with regard to separating the teeth of a lady of a strumous habit of body, twenty-eight years of age, for the purpose of obtaining sufficient room to insert fillings in their approximal surfaces. I suggested that I thought he would be running considerable risk by doing so. He was induced, however, to separate the incisors, cuspidati and bicuspids; in consequence of which, a diseased action was set up, which, in four years, resulted in the partial destruction of the alveolar process of the central and lateral incisors, and will, in all probability, occasion the loss of these teeth. I believe the judicious use of the file to be one of the most valuable operations in dental surgery, and, in a large majority of the cases, it has been my uniform practice where the patient exceeds twenty-one years of age, to file the approximal surfaces of the teeth preparatory to filling them. Much judgment, however, with regard to the difference of susceptibility of teeth is necessary, and when the proper precautions are used, I do not believe as much danger is to be apprehended as some suppose. Even in young persons, where I cannot separate the teeth with wedges, or gum elastic, I have sometimes felt myself called upon to use the file in order to secure a sound solid margin or wall around the orifice of the cavity of the tooth. Also, in cases where the decay has proceeded so far as to break away the enamel from the labio, or palato—approximal angles of the teeth, I believe filing to be the better method of separation.

Dr. E. J. Dunning wished to inquire of Dr. Harris, what were the symptoms which indicated that injury had been done by wedging, and how soon those symptoms appeared?

Dr. Harris. The symptoms are slight elongation of the teeth, inflammation and swelling of the margins and points of the gums which surround them, and ultimately a gradual loosening of the teeth, resulting from destruction of the sockets, depending altogether upon the patient's susceptibility to morbid impression. It is particularly necessary for the dental practitioner to be able to discriminate between these susceptibilities in different individuals. To illustrate my meaning, a scratch upon the hand, will produce in some, but a trifling inconvenience, but in others, it may give rise to extensive inflammation, ulceration and even gangrene. It is important, therefore, that every dentist who is in the habit of separating teeth with wedges, should be well acquainted with the indications of different constitutional susceptibilities.

Dr. W. H. Dwinelle inferred from the gentleman's remarks, that if he is to draw the teeth apart by elastic bands, he prefers to do it upon persons under the age of twenty years, and generally refrains from doing so on those over that age. In my own experience, I am induced to believe that the misfortune spoken of by the gentleman, was as liable to occur at one age as well as another. In the case of a young girl of thirteen, whose teeth were very irregular, I regulated them in the ordinary way, and the consequence has been elongation and inflammation, and unless I am more fortunate than I expect to be in her case, the destruction of one of the central incisors will ensue. I refer this case, however, to a morbid state of the system; the subject is scrofulous. I am not of the opinion that the age has so much to do with the result, but am inclined to think we are unable to anticipate fully and entirely what the result may be in any subject. I regard it as an experiment which will have its occasional failures as well as other operations in our profession.

Dr. Dwinelle heartily endorsed what Dr. Harris had said with regard to the practice of filing teeth. He said, also, that he had made it a point to be particular to polish the surfaces filed, and not to leave broken and velvet surfaces as so many infinitesimal nucleuses for the collection of foreign matter.

Dr. Harris. In examining this subject, we must do it as pathologists and not as mere mechanics. Previous to the growth of the body, the recuperative energies are more active than at a later period of life. Hence, it is, that an injury of lesion is more readily and perfectly repaired in young subjects than at a period of life subsequent to the age of twenty-five, consequently, there is a greater susceptibility to morbid impressions, provided, the state of the general health is the same. In the case mentioned by Dr. Dwinelle, as in many other cases, it may result in a series of disasters, so that it may not, in some cases, be safe before the age of twenty, to attempt to change the position of a tooth, though, as a general rule, in the treatment of irregularity, the sooner it is instituted, the better.

Dr. E. J. Dunning. I frequently use wedges for the purpose of separating teeth, more especially front teeth, but, I agree with Dr. Westcott, in preferring a thorough use of the file in treating caries upon the lateral approximating surfaces of bicuspides and molares. In treating these cases, I use the file very freely, making wide and permanent separations so shaped, if possible, that they will be cleansed by the constant action of the tongue, cheeks, saliva, &c.

In the treatment of the front teeth, however, the dentist must endeavor to secure, not only the health of the organs, but their beauty; while he remedies the defects caused by disease, he should never forget the importance of these organs to the expression and comeliness of the countenance, and while he is stern and unflinching in his adherence to correct principles of practice, he should not unnecessarily compromise symmetry of form.

There is one circumstance which has an important bearing on this subject of separating teeth which has not yet been mentioned. I refer to the habits of cleanliness of which we may observe the indications in the mouths of our patients. There are some cases in which thorough separations are imperatively demanded, in order to secure stoppings occurring in approximal surfaces or the parts immediately surrounding them from the action of decomposing agents, while in other cases, such precautions are unnecessary, these deposits being removed by the patient once, twice or thrice, in each day, as the case may require.

Delicacy of constitution—impaired health, acrid secretions from whatever cause or their opposites must enter into the account and assist the dental practitioner in determining the treatment.

Dr. Westcott, coincided with Dr. Dunning's remarks, that unless the dentist is governed by the circumstances of the patient, he will make but a poor operator. This saying, that lobelia is good for consumption, and steaming for a fever, may do for quack doctors, but not for us. We do not practice in that way. Let us take a particular case; for example, that of a child, whose teeth are closely crowded, but not uneven. In such a case where I can depend upon the good care of the teeth, my plan particularly for the last two years has been, first to separate the teeth by filing. When the cavities are of large size, I so separate the teeth as that they never will get together again. When the points only of the teeth touch, I have found the filing of the teeth to be bad practice, and if I must get space, I do it by wedges. In the case I have supposed, where the teeth are crowded, I first separate with the file about the thickness of a common visiting card; but do not carry the separation farther up than the apex of the gum. I then use wedges gradually so as to get sufficient room. Then I go through another process of filing in such a manner as to make the edge on the under side, shorter than the outside. This method of filing which can be easily accomplished after the teeth are separated as above described, fulfils two important indications, viz:

1st. It perfectly hides from *front* view, the fillings after they are inserted, although they may be plainly and distinctly seen from behind, when the mouth is open.

2d. The spaces thus left between the teeth, are such as may be with the least effort kept clean. If the proper care be taken in giving the right shape, and in leaving every point perfectly polished, little effort will be required to keep them free from food or other foreign matter. The action of the tongue and the fluids of the mouth will be generally sufficient.

We cannot work as easily upon children as upon adults, and unless we can get sufficient room, my own skill is not sufficient to perform a good operation more than half the time. I never operate but upon two cavities at a time; after completing which, I let them fall back to their natural position, and begin with two more. If I begin upon the lateral cavities, I next take the same on the other side of the mouth, so as to let the irritation subside before commencing another operation about the same spot. If the cavities are small, and the patient one whom I can operate on again soon, I defer the operation till the cavities are more fully developed. Of this, I may speak more fully hereafter, if time affords us an opportunity to discuss the subject of *filling;* with regard to the use of wedges, I accord with Dr. Harris, that there is a liability to chronic inflammation which may sometimes be lasting. Where we find the gum already absorbed with an evident predisposition to absorption with yellowish teeth, if the patient is more than twenty years of age, I conceive it hazardous to wedge, and if cavities are on the lateral surfaces, I should endeavor to get at them in some other way than wedging. If the patient could and would come often enough, so as to prevent the possibility of losing them from neglect, I should certainly leave the cavity until it was justifiable to file. As regards the manner of filing as I have already observed, I make the under portion of the teeth the narrowest. The front teeth should not be disfigured, but kept as near their normal shape as possible. In cases where the irregularity of the teeth is considerable, we are not obliged to file at all, the cavities being sufficiently exposed.

Were the manner of filing the special subject of discussion, I would make some suggestions upon the mode I have adopted in particular cases—here simply observing that by judicious management in performing this operation while the primary object may be to obtain space to enable us to fill the teeth, veiy much may be done to correct slight or even considerable irregularity of the incisors.

The general idea which I would bring forward, is this—if, for instance, the frontal incisors are irregular, the right over-lapping the left, unless the character of the decay contra-indicates it, I file mainly from the left incisor. The object will be readily understood, when we consider, that the edge left at any point of filing, is more prominent as we advance towards the centre of the tooth, and is hence in this way brought more nearly to correspond with the over-lapping tooth, whose contiguous edge was raised at the beginning above the tooth filed.

I have often succeeded in entirely overcoming slight irregularities in this way.

Dr. J. A. Cleveland, remarked, that in the early part of his practice, he was disposed to discard the file. Another dentist, who was then practising in the same community, had been in the habit of using the file freely for several years. I then differed with him, and cautioned him against its too free use. After some few years experience in practising upon the same patients, I discovered that I was in error, in cautioning my elder brother about going too far in the use of the file. Having continued ever since to practice in the same community, and having abundant opportunity to test the effects, I can say, that I have known probably three cases where the free use of the file was successful, to one where a very limited use was so. I can show filings where the bone has been exposed twenty years, and the teeth are sound. A worthy member of this Association, twenty-eight years ago operated upon many patients at the South, among whom, was my brother and one of his neighbors, upon whom I have since attended. I have lately examined their teeth, whose enamel was filed off so long ago, and I found them sound. They are both, however, attentive to their teeth. I now use the file much more freely than formerly. I have never used wedges or any other means. My method is, first, to introduce a very thin file, then I file away what I deem necessary for filling: I then reduce the edges where they come in contact to very small points by filing slightly from each side above and below, so as to bring the approximal points on as small spaces as possible. I file bicuspids and molars more than front teeth. I concur with Dr. Westcott, with regard to filing under; most of my fillings are put on the under-side. I have used the file in the manner described for fifteen years.

Dr. J. H. Foster. My treatment of the front teeth differs very much in respect to the mode of separation from that which I pursue with bicuspids and molars. I occasionally use wedges in separating the latter cases, but more frequently make free and wide spaces with a chisselled-shaped instrument, and then with the file complete the operation for making the space between the teeth level and regular, leaving it in form similar to the letter V. I use the file between the front and lateral incisors and the cuspidati, only so far as to secure the separation which they require after the operation of filling is performed, except in those cases where the parieties of the cavities are so thin and weak as to require that more should be removed. So much injury has been done by the indiscriminate and reckless use of the file, for which the poor persecuted instrument has borne the obloquy which should have attached to the hand that used it, and yet it is powerful for good as well as evil, if treated kindly, that I have been more cautious in handling the file than any other instrument. With respect to wedging, I prefer this course of treatment, and as a general rule am governed by it in separating the front teeth. I use India rubber in preference to wood, because I think it less irritating. I have three or four different thicknesses, which I insert successively in all cases in which it is possible. Wait until the inflammation consequent upon the pressure has entirely passed away, before performing the operation of filling; the severe inflammation spoken of has never come under my notice in any case, and must be attributed, I think, to too great a degree of pressure upon the teeth in the commencement of this treatment.

Dr. Robert Nelson. If the patient be young—say under twenty—and the cavity small, I press the teeth apart by means of fine linen or cotton cloth, drawn between them—increasing the folds as circumstances may direct.

Should the decay have progressed so far as to make it necessary to remove a portion of the tooth, in order to give form and strength to the sides of the cavity, but not sufficient to give the requisite space for filling, I remove some of the soft bone, and fill the cavity and intermediate space with cotton: the cotton, by absorbing the fluids of the mouth, swells and presses the teeth apart with a gentle, though irresistible force.

I have used various articles for this purpose, but find none to possess the advantages of cotton; it is not only easily applied and retained in the cavity, but it also, while it separates the teeth, presses the *gum* from the margin of the cavity—an office of the greatest importance.

I may here add, that although I have been in the habit of pressing teeth apart for the last fifteen years, yet have I never seen the slightest evil consequences resulting therefrom.

For the back teeth, my *general* practice is to separate them with a chisel, or file; and I feel assured from my own experience in a practice of many years, and from observation on the practice of others, that these instruments may be used with perfect safety by the skilful operator; indeed I have always observed that those who have been most successful in their practice, have used them with the greatest freedom.

When teeth approximate at the points, the tendency of the wedge is towards the gum, which produces the irritation described by Dr. Harris. It is my practice in such cases, to pass a thin file between, which gives room for the wedge, and prevents the crowding towards the gum.

Dr. Westcott thought that the difference in the kind of wedges used, arose from one's making a right use of one kind, and seeing a bad use of the other. He had several years used rubber principally, but now prepared fine wood, perhaps if he had adopted the assorted India rubber at first, he might have approved of it better. He liked wood also, because it will retain the teeth in their places. By shaping the plug like an hour glass, it can be worn several days till the soreness gets out, which he was unable to do with gum elastic.

Dr. Harris said he had tested the relative merits of both wood and gum elastic, having used them indiscriminately for a number of years. To separate teeth, a certain amount of inflammation is necessarily excited, by the pressure of the organs against the alveolo-dental periosteum, and the greater the pressure the more active, as a matter of course, will be the inflammation. When the pressure is exerted very slowly, the pain will be slight, causing the patient but little inconvenience. But, as I have before stated, a certain amount of inflammation is necessarily produced, and as the alveolo-dental periosteum becomes inflamed, a chemical fluid is poured out, which breaks down the surface of the alveolar wall, against which the tooth is pressed, and it is partly in this way, and partly by the yielding of the alveolus, that the tooth is moved. It is true, the irritation may, and often is, very greatly increased by the pressure of the interposed substance against the point of the gum.

Dr. Harris stated that he had seen teeth which had been filed thirty-five years, that were still sound, yet many teeth, thus filed, may have decayed on account of the greater susceptibility of the organs to the action of the causes that produce the structural alteration. This should always be considered, as well as the patient's habits of cleanliness, with regard to the teeth. Some teeth may be able to resist the action of the usual causes of caries, throughout the whole period of life, if the filed surfaces be kept thoroughly and constantly clean, but teeth of a soft texture will ultimately be decomposed. But as I have elsewhere given my views upon the manner of performing this operation, as well as upon its utility, it is unnecessary for me to say more upon the subject.

Dr. J. B. Rich. In the early part of my practice, I was informed of Dr. Parmly's method of separating the teeth by means of wedges of wood. I availed myself of this method whenever I met with an opportunity to do so. The result was by no means satisfactory. In several cases, so great was the inflammation superinduced, that the teeth lost their vitality, consequently I abandoned the use of wood for that purpose.

I next heard that Dr. Brewster had used India rubber for separating the teeth. My early experiments with this substance produced nearly as much inflammation as my experiments with wood. I continued, however, the experiments, and in two cases succeeded in separating the teeth with scarcely any inflammation. This encouraged me to pursue the experiments, which I did, with the following results: I found that by carefully graduating the sizes of the pieces of rubber used, the teeth might be separated a considerable distance without producing much inflammation. I cut the Indian rubber to the requisite thickness at the time it is to be applied, which can easily be done with scissors. For some time past, I have prepared the rubber for use, by boiling it in water, until it becomes quite soft. When thus softened, it is superior to any substance, with which I am acquainted for this purpose. When the rubber is thus made soft, the force is applied so gently, that there is very little, if any inflammation to be apprehended from its use.

It takes, I am free to confess, longer time to separate the teeth by means of boiled rubber, than either by wood or by the rubber unboiled. But as it is of the utmost importance to prevent inflammation of the periosteum during this operation, I less regret the delay. The rubber after boiling, is quite as elastic as it was before; but its mechanical force is considerably diminished. If I separate the teeth for the purpose of filling on the approximating surfaces, and wish them afterwards to resume their original position, I reduce the thickness of the rubber, day by day, until they shall have returned to their original place. It is quite as desirable that the teeth should come together gradually, as it was desirable that they should be separated gradually. This has been my practice, and the results have fully answered my expectation. I should be happy, however, to be instructed by the suggestions of others upon this subject,—as to me, it has ever been an exceedingly interesting one.

Dr. Foster inquired of Dr. Rich, if he could always manage to get the soft rubber between the teeth?

Dr. Rich replied, that the soft rubber would be introduced between the teeth much more easily than the hard. When it was found difficult to introduce the rubber, on account of the teeth being close together, it was his (Dr. R's) practice to pass floss silk waxed between them and to allow it to remain between a few minutes. After which the rubber might be introduced without difficulty. As it regards the operation of filing it was Dr. R's practice to file or cut away the surfaces until the edges of the cavities were firm enough to bear the pressure of filling. He would not leave a cavity to grow larger before it was filled, nor would he hesitate to file away the surface on account of a cavity being small. If decay exist in any part of a tooth, and that decay cannot be cut or filed away without disfiguring the tooth, it ought to be filled at once. The smaller the cavity, the greater would be the service rendered to the tooth by filling. If the decay be left in order that the cavity may become larger, the caries may extend in the direction of the cavity of the nerve, instead of spreading over a superficial surface.

Dr. Westcott said that he had not yet met with a case where he could not determine whether the operation of filing should be performed at once, or left for the cavity to increase in size. If there was any doubt about it, it was better to operate early than to leave it till the nerve was exposed. With regard to the material used for separating the teeth, Dr. W. had seen an instance in which white wax had been used by a lady to supply the place of a lost tooth, and it had, by continual renewal, gradually increased the space between the teeth so as to leave ample room for another tooth: she had worn the wax for eight years, and the gum was perfectly healthy.

Dr. Dwinelle thought Dr. Rich was rather too particular and nice in the matter of separating teeth, and of reducing them back again. The necessity of gradually reducing them back, was not very apparent to him, because they would naturally return to their places gradually.

Dr. Dunning said that he had used India rubber but little, having found it exceedingly difficult to make it retain its place between the points of teeth that approximated very closely. In such cases he was in the habit of using a very small quantity of cotton, for the purpose of commencing the separation, which was completed by wedges of wood. The cotton is introduced by the aid of a thin and very smooth instrument of steel, which forces it to the desired position between the teeth.

Dr. J. Parmly had not found any particular difference in the materials used, except when greater pressure was produced. He had performed a great many operations of separating the teeth, and had never found them to result in the death of the teeth. By a certain pressure with a steel instrument, you may destroy the tooth, and it will not show till perhaps a month after, but I have never seen a tooth destroyed by wedges. In moving the tooth, its connection with the nerve cannot be destroyed. In no case can the teeth be moved, as far as my experience goes, without some pain. If the amount of pressure is produced which will separate the teeth, some pain must be produced. I have known several cases where the teeth have separated without any pressure. In one case where I extracted a tooth, I found two-thirds of the whole membrane destroyed, and the outside adhering closely upon drawing the tooth out.

Dr. Foster cited a case of a young lady of thirteen years of age, whose upper front incisors projected very far out beyond the under.

The space between these teeth had become very wide, and after regulating them and confining them in their proper position, he found the antagonizing teeth did not touch them upon their inner surfaces, nor was there any evidence that they had ever done so. The gradual departure in this case from the natural position, had been caused by a constant habit which she indulged in of keeping her thumb in her mouth, and exerting continual pressure upon these teeth outward.

Dr. Rich. Few will deny that it would be mal-practice to force the teeth suddenly into the position desired. In my opinion it is the wiser practice to move them in the slowest manner possible, nor do I imagine that I shall be found singular in this opinion.

Many practitioners entertain a notion that the teeth cannot be separated without pain. Experience has taught me the reverse, provided always the separation be effected gradually and gently. If during this process of separating, where I see the teeth every day, and am informed by the patient that there is no pain, and if upon examining the parts, I detect no inflammation, it is to my mind the best possible evidence that my theory and practice are correct; more especially, when this state of things occurs again and again in different individuals. One incontrovertible fact outweighs all the improved theories that can be advanced.

I have often moved teeth considerable distances in young persons, when remedying irregularities without pain to the patient. In one operation of this kind, which was performed without pain, I was obliged to move a central incisor nearly a quarter of a circle before it was brought within the line of the arch. The patient was a lad 15 years old, and when I first saw the case, the tooth projected out of the mouth, so that the lip was raised by it considerably. Still the operation was performed with so small an amount of inflammation or inconvenience, that after the operation was completed, the boy said to his father—"I do not think it required much skill to fix my teeth; I could have done it myself if I had had a piece of Indian rubber and some gold wire." I can conceive no greater compliment to a practitioner, than was conveyed in this assertion, that the skill of the dentist could render a most difficult and generally painful operation so apparently easy that the child should have thought that he could have done it himself.

Good evidence that the teeth can be moved from their original position without pain, may also be found in the fact, that the teeth, particularly the incisors, often change their position of themselves moving to the right or left and leaving large spaces between the teeth or projecting out in front. If we question the individuals with whom this has occurred, we shall be told that the change has taken place gradually and to themselves imperceptibly. Repeated observations have convinced me these alterations in the position of the teeth are brought about by pressure of the antagonizing teeth and occur where the whole force of the jaw is brought to bear upon one tooth either by the loss or wearing down of the back teeth or by the swelling of the periosteum of the displaced tooth. In order to restore teeth which have been thus displaced to their proper position, I have always found it necessary to commence by filing the antagonizing surfaces so that they could on longer come into contact.

Dr. Foster thought with Dr. Rich, that the softer the substance used, the less would be the irritation. He had some cases in remembrance of patients who always separated their own teeth, and preferred doing so, succeeding quite as well in making wide spaces in less time, and as they said, with less pain, by compressing a pledget of cotton between the teeth.

Dr. Nelson. It is astonishing how slight a pressure will separate the teeth. With regard to the teeth returning again to their place, Dr. Rich's practice differs from mine, which has uniformly been to let the teeth return again as soon as possible. If I separate them, I immediately apply a force to the lateral sides. If you draw a piece of rubber between your teeth, it necessarily produces pain; if you remove it, the pain subsides. With regard to filing, I am satisfied that in many instances it is the only mode of saving the teeth. It is bad practice, however, to leave the filed surfaces touching each other. I know a lady whose teeth were filed quite half away twenty-five years ago, and there is no sign of decay. The practice of separating by wood does not commend itself to me, because the wood seems to expand rapidly from the moisture, and produces a great deal of pain. Rubber causes less pain; cotton acts slowly, and has a happy effect. If the cavities are large, extending under the gums, the points of the latter are pressed away for the time being, and gives the operator an opportunity to fill.

Dr. Dunning thought cotton was only preferable when the surfaces approximate so closely and the edges were so sharp that wedges would be likely to break in introducing them.

Dr. Rich described his method of preparing India rubber for separating the teeth. I use the natural or white rubber, and prepare it for use by boiling it eight or ten hours. The rubber requires to be kept moist and warm after boiling, or it will become hard again in a few hours. Moreover, this process changes the color of the rubber, making it nearly white, so that it does not disfigure the mouth by presenting a dark mass between the teeth.

Dr. Westcott wished to be understood as to what he had said with regard to leaving cavities to increase, so as not to be obliged to file away too much. No matter how small the cavity was, if it was deep, it ought to be filled immediately. Not unfrequently the cavity was so slight as not to warrant the disfiguring of the teeth by filing. He had in his mind such cavities on the convex surfaces of molar teeth. A week since he was called on to fill such a cavitiy between the wisdom tooth and second molar. Upon examining the cavity he told the patient to let it go for the present, and by and by it could be filled without as much filing as now. In regard to small cavities in the front teeth, Dr. W. uniformly separated by wedges, preferring to have one uniform surface.

Dr. Dunning considered the subject of practical dentistry of thrilling interest, and expressed his gratification for the rich treat he had enjoyed, in common with the rest, in the interchange of thoughts on this important subject.

Dr. Harris. As the operation of separating teeth with wedges of wood, or some other sufficiently hard substance, originated with the President of this Society, if I am correctly informed, it would give me pleasure, as I doubt not, it would all the other members of the Association, to hear his views upon the subject.

The afternoon being exhausted, the further discussion was adjourned till the next day.

[*To be Continued.*]

*Philadelphia, March 23d,* 1850.

Prof. Chapin A. Harris:

*Dear Sir:*—I send you through my friend, Dr. E. Townsend, ***a tooth,*** which possesses more than ordinary interest. In February, Mr. F., called upon me to have an aching tooth examined; my attention was called to a molar tooth, right side superior maxillary, which I examined closely, and could find no marks of disease about it, but in front of it a bicuspid which had considerable periosteal inflammation, I advised him to have removed, believing it would rid him of all pain. Mr. F., returned to me again within twenty-four hours, with as much pain as ever, and insisted upon the extraction of the molar tooth as that was the seat of pain; I again examined the tooth more closely if possible than at my previous examination. I could not find any point which would lead me to suppose that the tooth ached, more than his word, which would not be a guide sufficient to extract a tooth. I desired him to postpone the operation for a day or two, thinking the pain would pass off, but could not prevail on him to defer it; he said he would not suffer as much as he had again for all the teeth in his head.

When I found him so determined, I, as a matter of course, consented to extract the tooth. This done, I examined it again to see whether I could now find the cause. On examination, I found a small opening in the palatine root which explained the whole mystery.

I have come to no conclusion as regards the cause of decay in so singular a situation. The patient informed me that I had plugged the said tooth about five years before. I should be happy to have your views on the subject, as I am yet but a student in dentistry.

And I am most respectfully, your obedient servant,

*No.* 252 *Walnut St.* C. C. WILLIAMS.

---

### From our Paris Correspondent.

Paris, *June 27th*, 1850.

Our budget of correspondence on the present occasion must necessarily be of a very general and miscellaneous character. Nothing of particular interest has recently occurred; there are, however, some events, which, from their historical importance, deserve to be noticed.

The scientific world has been called upon during the month of May, to mourn the loss of two of its most distinguished lights. M. Gay Lussac and M. de Blainville, at the end of long and illustrious careers, and almost at the same moment, have been called from the the scene of their labors and their honors. Both of these gentlemen belonged to the Faculty of Sciences of the University of Paris, and lectured at the Garden of Plants. M. Gay Lussac has a reputation of nearly half a century's standing. His history has been the history, during the same period, of the science he has so assiduously and so successfully cultivated. His name is attached to many important discoveries in chemistry, and by extending its application to the purposes of agriculture and the arts, he has rendered the world at large essential service.

M. de Blainville was the successor or the *continuateur*, as the French significantly express it, of the great Cuvier. He was devoted to his favorite pursuit to the very last moment of his life; indeed, he may be said to have died a martyr to it. He occupied the chair of Comparative Anatomy, at the Garden of Plants and at the Sorbonne.

The enthusiasm of these distinguished men was truly astonishing.

Their places under ordinary circumstances, would be hard to fill, but thanks to the enlightened liberality with which the French government patronizes and fosters science, they will probably not be suffered to remain long vacant.

An event much talked of at the time, was the meeting of the Paris Medical Society, on the 27th of April last. This meeting was convened in honor of Professor Simpson, of Edinburg, then on a visit to Paris. Professor Simpson is a gentleman of deservedly high reputation in his department of the medical profession, but is, perhaps, better known to the readers of your Journal, as the discoverer of the anaesthetic power of chloroform. The experience of the mechanical world, as to the effects of ether, had not in the opinion of Professor Simpson, proved satisfactory. Whether from accident or from causes inherent in the very nature of this agent, it seemed liable to very serious objections, and yet its partial success encouraged him in the hope and endeavor to find some substitute which would completely answer the purpose desired. The possibility of neutralizing or even alleviating the suffering of patients obliged to submit to the excruciating pains of surgical remedies, rendered almost certain by the wonderful effects attributed to ether, had awakened the attention of medical men to the importance of the subject. Professor Simpson discovering at once its important relation to his own branch of the profession, was induced to give the matter a thorough examination. Regarding the experiments with ether as having established the fact of the applicability of anaesthetic agents, he was anxious to find something more safe and certain in its effects; he was at length fortunate enough to discover in chloroform a substance fulfilling in all respects, the conditions required. The Professor adduced a great number of interesting statistics in illustration of the benefits of chloroform. Without saying any thing positively in depreciation of ether, he insisted strongly upon the merits of the agent of which he claims the honor of discovery, and of the signal success with which its use has thus far been attended. He considers it, when properly administered, free from all danger. In cases where accidents have accompanied its use, he has been able to trace the cause to abuse, impurity, or some other source foreign to the nature of the substance itself. The same remarks might perhaps be made with equal truth, with respect to ether. No doubt it has suffered from the imprudence and ignorance of those who have undertaken to administer it, and perhaps, if it were always applied with the same degree of caution, and if equal pains were taken to use it only in a pure state, its chapter of accidents would dwindle down to the same dimensions as that of chloroform.

Dr. Simpson advocated the use of chloroform in case of accouchment. He observed that he had never witnessed any bad effects to the body or mind of the child born while the mother was under its influence, and his experience had made him acquainted with no instances of puerperal insanity on the part of the mother resulting from this cause.

The purity of the chloroform employed is of essential importance. That of the highest specific gravity is the best; its odour is mild and not disagreeable. When nausea, headache, stupidity or coughs occur, they may be attributed to the presence of other substances. Chloroform in its purest state is manufactured in Scotland by Duncan & Lockhart, while that used at Paris, as the Professor had had occasion to verify at their cliniques, was very much adulterated.

The quantity used is not so material as the manner of applying it. The only apparatus required is a simple pocket handkerchief held in such a manner before the mouth and nose as to allow a free admixture of atmospheric air. Prof. Simpson had been in the habit of using half an ounce at a time. In surgery practice larger doses are given than in midwifery—in the latter case it is administered *only* during the pains of labor.

Since my communication of last December, in which I recorded the testimony of my own judgment and experience as to the use of amalgams for filling teeth, I have observed that the inventive genius of the profession has not been idle. At least three candidates have made their appearance in the field in this country, and singularly enough, they all base their claims upon the exclusion of mercury. The first of these came to my knowledge a short time after the public acknowledgment of the ill success of my own experiments. About the 25th March last, I was informed that a happy idea had occurred to a chemical gentleman of Paris, who immediately resolved to turn it to profit if possible; his success, however, I believe, has not transpired yet. The third case has but just come under my notice. In at least one of the inventions alluded to, gutta percha is made to play the part of mercury.

Without having the vanity to take to myself the credit of bringing about a revolution of opinion and practice in regard to mercurial preparations, I cannot help being gratified at the fact as an indication of the prevalence of sounder views in the profession on this subject in this country.*

THOMAS W. EVANS.

### *Second Annual Meeting of the Society of the Alumni of the Baltimore College of Dental Surgery.*

The opening address to the Society of the Alumni, at their Second Annual Meeting, was delivered in the Saloon of the Baltimore College of Dental Surgery, by Dr. E. Townsend, of Philadelphia, on the evening of the 27th of March, and as our readers have already had an opportunity of perusing this most chaste and eloquent production, it is unnecessary for us to speak of its merits.

The next day at 3 P. M., the Society again convened for the transaction of its regular business, and after being called to order by Dr. H. COLBURN, President, the Recording Secretary read the proceedings of the last meeting. The chair then appointed Drs. P. H. AUSTIN, L. S. BURRIDGE and M. J. CHERRY, a committee of arrangement. After which, the following resolutions were offered and adopted by the Society:

* Here the attention of chemists and others is being directed to finding something free from mercury.

1. *Resolved,* That the Treasurer of the Society be instructed to call upon delinquent members for the payment of their dues.

2. *Resolved,* That all committees appointed by this Society to prepare papers, or report upon any subject connected with dental science, shall consist of but one member; and that Article 9, Section 2, of the By-Laws, be thus amended.

3. *Resolved,* As an amendment to Article 7, Constitution, that the Annual Meetings of the Society be held at such time during the week preceding the Annual Commencement of the Baltimore College of Dental Surgery, as the Secretary with the advice of the President, shall, on the first of January, deem most suitable.

4. *Resolved,* That in the event of the resignation or death of any officer or member of committee, it shall be the duty of the Corresponding Secretary to report the same to the President, who shall have authority to fill the vacancy, until the next Annual Meeting.

5. *Resolved,* That the thanks of the Society be tendered to Dr. E. Townsend, for the very eloquent address delivered before them last evening, and that a committee of two be appointed by the chair, to wait upon Dr. Townsend, and request a copy for publication:

Whereupon, Drs. L. S. Burridge and M. A. Hopkinson, were appointed for that purpose.

6. *Resolved,* That the thanks of this Society be tendered to the Faculty of the Baltimore College of Dental Surgery, for the use of the rooms so kindly offered them.

7. *Resolved,* As an amendment to 2d Section, Article 3d, Constitution, that the under-graduate members of this Society be eligible to the duty of preparing such papers and reports as may be appointed by the Society.

8. *Resolved,* That the Corresponding Secretary be empowered to enrol as members of this Society, any graduate of the Baltimore College, who shall apply to him for admission, upon payment of the initiatory fee of three dollars, to the Treasurer.

After the adoption of the foregoing resolutions, several of the under-graduates were elected active members of the Society, as was also Dr. C. G. Davis. The Society then elected Drs. A. Westcott, of Syracuse, N. Y., L. S. Parmly, of New Orleans, J. A. Cleaveland, of Charleston, S. C., Wm. H. Dwinelle, of Cazenovia, N. Y., James Robinson, of London, and Thomas W. Evans, of Paris, honorary members.

The chairman of the committee for the selection of Questions for scientific investigation, now presented five, from which the Society selected the following:

*First,* The comparative merits of single mineral teeth and blocks, with the best method of attaching the latter.

*Second,* The different forms of suction plates—their merits relative and absolute.

*Third,* When and where is the extraction of teeth admissible in the correction of irregularity, and in crowded dentures?

After the transaction of some other business, Dr. P. H. Austin read an address from Dr. James Robinson, of London, which we publish in the present number of the Journal, feeling assured our readers will be as much gratified to peruse it as we did to hear it delivered. Although it presents a lamentable picture of the state of the profession in England, it at the same time furnishes us the gratifying assurance that it contains many men of high merit and distinguished ability.

The Society now, after directing the Corresponding Secretary to express to Dr. Robinson the gratification which they derived from listening to his address, and request permission to publish it, proceeded to the election of officers for the ensuing year.

J. H. A. Fehr, M. D., D. D. S., President.
Charles W. Ballard, M. D., D. D. S., Vice President.
C .O. Cone, M. D., D. D. S., Treasurer.
P. H. Austin, M. D., D. D. S., Corresponding Secretary.
H. Colburn, M. D., D. D. S., Recording Secretary.
C. O. Cone, Standing Committee.
C. W. Ballard, Committee on Dental Practice.
P. H. Austin, Committee on Mechanical Dentistry.

The above officers having been elected, the Society adjourned.

*Balt. Ed.*

---

*Dr. C. S. Brewster,* who, for the last seventeen years, has resided in Paris, in the enjoyment of a very extensive and lucrative practice, is now on a visit, with his beautiful and highly accomplished lady, to the United States. They expect to return to Europe, however, in the early part of October. *Balt. Ed.*

---

Dr. B. A. Rodrigues, Surgeon Dentist, of Charleston, S. C., and formerly a pupil of Dr. Brewster, passed through Baltimore a few days since, on his way to Europe. He took passage on board the steamship Atlantic, and expects to be absent about three months. We wish him a pleasant trip and safe return. *Balt. Ed.*

---

*Gold Foil.*—Drs. Blanding & Avery, of Columbia, S. C., have recently commenced the manufacture of their own gold foil, and from a specimen of a few leaves of No. 8, they were so kind as to send to us, which we have used, we have no hesitation in pronouncing it vastly superior to the foil of very many of our manufacturers. Except from the manufactory of Charles Abbey & Son, of Philadelphia, we have rarely, if ever, used foil of a better quality.—*Balto. Ed.*

---

*American Society of Dental Surgeons.*—The next meeting of this Society is to be held at Saratoga Springs, commencing August 13th. As business of great importance will come before the Society at this meeting, we hope it will be well attended, and yet, as the place at which it is to be held is so remote from most of the members, we fear that but few will be present. We trust a more central point will be selected for the future meetings of the Association.

---

*Dr. E. Townsend's Address.*—The Address delivered by Dr. E. Townsend, of Philadelphia, to the Graduating Class of the Baltimore College of Dental Surgery, at the last annual commencement, has just been published, and will be read, we know, by all into whose hands it may fall, with pleasure. It is beautifully written, and contains much good advice. We hope that others as well as those to whom it was more particularly addressed, may profit by it.

---

*Harris's Principles and Practice of Dental Surgery.*—The third edition of this work having been out of print for several months, the publishers, Messrs. Lindsay & Blackiston, have recently issued a fourth and greatly enlarged edition, with forty-five new illustrations.

*Valedictory Address.*—By request of the Faculty as well as of the graduates of the last Annual Commencement of the Baltimore College of Dental Surgery, the chaste and ably written Valedictory Address of Dr. S. P. Hullihen, of Wheeling, Va., has been published. We have seldom, if ever, listened to, or read a more appropriate or abler production of the kind, than this. We wish it could be read by every dentist, and every physician throughout the length and breadth of our land, and had not a copy already been sent to every one of our subscribers, we certainly should re-publish it in the Journal. *Balt. Ed.*

*Completion of the Tenth Volume of the Journal.*—This number completes the tenth volume of the American Journal and Library of Dental Science, and if all the subscribers who are in arrears, would promptly remit, on the receipt of it, the amount of their indebtedness, the publishers would commence the Eleventh Volume with renewed energy and zeal, making the work vastly superior to what it has ever yet been. Much of the time which the writer would have been glad to have devoted to the preparation and procurement of materials for its pages, has been necessarily occupied in the management of the business connected with its publication, and rendered indispensible by the omission on the part of a large proportion of the subscribers to pay their subscriptions. Now, if each subscriber would feel that the continuance and scientific and practical character of the publication depended upon the promptness with which he paid the amount of his own individual subscription, the writer would be saved a vast amount of labor.

Many of the subscribers to the Journal either pay in advance or promptly whenever they are notified of their delinquency, and we do not believe that any fail to do so with a design of defrauding the publishers. On the contrary, we are inclined to attribute their negligence to carelessness and forgetfulness; each one supposing that the amount which he is owing is but small, and that it cannot be a matter of much importance to the publishers whether he pays this week or next, thus neglecting it from week to week, and year to year, until the amount of his subscription runs up to twenty or thirty or forty dollars, the payment, from the increased amount becoming every year more and more inconvenient.

Now, when we tell our delinquent subscribers that they are owing the Journal between three and four thousand dollars, we feel assured they will not any longer neglect to send us the amount of their arrearages. We sent a bill a few weeks ago to each one, and a few responded promptly to the call which we made on them, and we hope that those who have not yet done so, will not neglect to do it any longer.

The delinquency of subscribers is a subject to which we allude with great reluctance, and would never do so if it could be avoided.

*Balt. Ed.*

